# The Purpose of Food Processing and Ultra-Processed Food: the Potential, Pitfalls and Path Forward for Public Health

**DOI:** 10.1007/s13668-026-00775-z

**Published:** 2026-06-06

**Authors:** Samuel J. Dicken

**Affiliations:** 1 Centre for Obesity Research, Department of Medicine, University College London (UCL), London, UK; 2 Behavioural Science and Health, University College London, London, WC1E 7HB UK; 3 University College London Hospital (UCLH) Biomedical Research Centre, National Institute for Health Research, London, W1T 7DN UK

**Keywords:** Ultra-processed food, Diet, Processing, Public health, Nutrition, Obesity, Diet-related disease

## Abstract

**Purpose of Review:**

The Nova classification considers the purpose and degree of food processing to capture how recent changes in the food system may link with health. Its value for understanding diet-disease relationships is debated. However, reviews typically focus only on its value, or its limitations. Furthermore, by discussing the degree of processing only and not the purpose, or discussing processing in general, reviews of limitations do not address pitfalls of the Nova classification. This conflict and confusion stalls progress to improve public health. Therefore, this review aims to provide a balanced understanding, interpretation and utilisation of the Nova classification and purpose of food processing, with a critical evaluation of its potential value, of pitfalls largely overlooked to date with recommendations to avoid unintended consequences, and constructive reflections on a path forward.

**Recent Findings:**

The Nova classification offers a framework linking upstream socioeconomic factors with downstream properties of available food, complementing existing food-based dietary guidance to improve access to healthy diets through food systems transformations. However, its practical application, narrative of intrinsic harm of ultra-processing, usage to guide diets in isolation, and overemphasis on mechanisms with weaker evidence are pitfalls that, whilst avoidable, can have unintended consequences.

**Summary:**

There is agreement on the need for food systems transformation to ensure access and affordability to healthy diets for all. For public health, the purpose behind food processing provides an understanding of systems drivers of food production and accessibility. This consideration can be reflected in policy to improve access to healthy diets, without necessarily explicit use of the Nova classification.

## Introduction

Suboptimal diets are a major cause of global morbidity, and overtaking tobacco smoking as the leading cause of mortality [1]. In particular, besides excess sodium intake, diets low in healthy food result in greater disease burden than diets high in unhealthy food [1]. But, millions worldwide face barriers in accessing healthy food [2]. A key underlying factor has been the recent change in the food environment towards a globalised, industrialised and commercialised system [3]. Whilst policies have been introduced to address suboptimal diets, these focus on individual-level action, with minimal impact in halting the rise in diet-related disease [4, 5]. Recently, attention has turned towards dietary patterns and upstream determinants of food availability, accessibility and affordability [3]. This approach considers not just *what* our food is, but *why* it is.

### Food Processing

Food processing (any procedure changing the natural state of food) has existed for generations [6], where the primary purpose of processing has been to ensure food is edible, safe, palatable, durable, and nutritionally improved (e.g. fortification) [7]. Farmers would cut, grind and preserve crops and livestock to create food and drink to eat and/or trade locally. However, since the industrial revolution in the 20th century, drivers to further repurpose crops and livestock have facilitated a food system transformation [8]. Following World War II, economic incentives enabled food corporations to increase food production to meet the demands of rapid population growth [8]. Coupled with technological innovation in food processing, industrialisation and globalisation of the food system [9], corporations were able to create a plentiful, affordable, safe and long-lasting supply of energy, with significant financial success [8]. These economic incentives provided significant benefits to global food security, saving billions from starvation [9, 10]. However, they still remain, and continue to shape the food supply [11].

Several classifications aim to understand how this recent food system transformation and revolution in food processing may link with diet-related disease [12]. These predominantly classify foods based on the physical degree of change [12], with the lowest degree considered unprocessed/minimally processed food (MPF), and the highest degree considered highly-processed or ultra-processed food (UPF). But, food processing can also describe the nature (e.g. change properties or add ingredients), place (e.g. home or factory), and importantly, the purpose (e.g. for safety, for edibility, for profit) [12]. Unlike other classifications which define food based on the degree of processing only, the Nova classification intends to capture how the primary purpose of processing links with food properties and health [13]. There is purpose behind any food processing. In the Nova classification, the primary purpose of UPF is financial gain, which has been taken to the extreme as the sole driving factor shaping the product to maximise profitability. The current food system is now increasingly dominated by UPF [3, 14] (Table 1).

**Table 1:** Detailed overview of the Nova classification by primary purpose, degree of processing, formulation and identification

	Primary Purpose of processing	Degree of processing	Processes	Formulations	Includes industrial processing?	Can be fortified with vitamins and minerals?	Can contain additives?	Identification
Minimally processed food	To remove inedible or unwanted parts, extend shelf life, and make their use in a culinary preparation easier or more diverse.	May undergo some degree of physical processing, e.g. coffee grinding, flour milling, fruit juice blending.	Drying, crushing, grinding, fractioning, roasting, boiling, pasteurisation, refrigeration, freezing, placement in containers, vacuum packaging or non-alcoholic fermentation.	One or more minimally processed foods.	Yes	Yes	Yes, can infrequently contain functional additives to extend shelf life, protect original properties or prevent proliferation of microorganisms [13]. Does not contain additives with only cosmetic functions.	Fruit and vegetables, grains (e.g. rice, corn, wheat) legumes, starchy roots (e.g. potatoes), fungi (e.g.mushrooms), meat, poultry, seafood, eggs; fresh orpasteurised milk, plain yoghurt, fruit and vegetable juice, flakes and flours (e.g. wheat, oats), nuts and seeds, herbs and spices, tea, coffee, and water. Containing no processed culinary ingredients, industrial ingredients, or cosmetic additives.
Processed culinary ingredients	To create ingredients for seasoning and cooking meals prepared from scratch with minimally processed food. Usually not consumed by themselves.	May undergo some degree of physical processing, e.g. oil extraction.	Obtained directly from MPF via pressing, centrifuging, refining, extracting, or mining.	One or more processed culinary ingredients	Yes	Yes (e.g. iodised salt)	Yes, can contain functional additives to extend shelf life, protect original properties or prevent proliferation of microorganisms [13]. Does not contain additives with only cosmetic functions.	Vegetable oils, solid fats (e.g. butter, lard), sugars (e.g.sugar and molasses from cane or beet, honey, syrupfrom maple), salt (mined or sea salt), salted butter.
Processed food	To increase the durability and long-term safety of minimally processed foods and make them more enjoyable by modifying or enhancing sensory qualities.	May undergo some degree of physical processing, e.g. cheese production.	Preservation methods including canning, bottling and non-alcoholic fermentation.	Basic combination of minimally processed foods and processed culinary ingredients.	Yes	Yes	Yes, can contain functional additives such as preservatives and antioxidants that prolong duration, protect original properties, or prevent proliferation of microorganisms [13]. Does not contain additives with only cosmetic functions.	Relatively simple manufactured food products: tinned fish in oil, smoked, dried or salted meat, tinned or bottled vegetables or legumes in brine, artisanal cheese and bread, fruit in syrup, and salted nuts.
Ultra processed food	To generate profit [13], [19]. Profit maximisation achieved by creating highly appealing (palatable with optimal sensory properties) products, using the cheapest ingredients with extended shelf-life. This makes products as accessible and appealing as possible, allowing widespread distribution and supported with aggressive marketing. New products can expand to create additional eating occasions, and displace less processed foods [19].	May undergo an extreme degree of physical processing.	Requiring sophisticated equipment and technology such as extrusion, moulding and pre-frying.	Multi-ingredient formulations manufactured by deconstructing foods into component parts (for example, oils, starches and protein isolates), modifying them (e.g. with enzymatic processes) and recombining them with cosmetic additives (e.g. flavours, flavour enhancers, colours, emulsifiers, sweeteners, thickeners, and anti-foaming, bulking, carbonating, foaming, gelling, and glazing agents). Sugar, oils, fats, and salt (generally in combination and in higher amounts than in processed foods) and food substances of no or rare culinary use (e.g. high fructose corn syrup, hydrogenated oils, modified starches, and protein isolates).	Yes	Yes	Yes, contains functional additives, but uniquely also contains cosmetic additives (used to hide any undesirable sensory properties from the ingredients, processes or packaging used, or make products more attractive tosee, taste, smell and/or touch, without functional use) [32].	Operationally distinguishable from processed foods by of food substances of no culinary use or of additives with cosmetic functions in their ingredientslist [19]. Whilst industrial ingredients and cosmetic additives may or may not directly impact health in and of themselves (discussed in Pitfalls), their presence isused as a marker of a product undergoing optimisation for profitability in the ultra-processed business model and ultra-‘profitable’ cycle [19]. Soft drinks, sweet/savoury packaged snacks, chocolate, candy, ice-cream, mass-produced packaged bread, margarine, biscuits, pastries, cakes,breakfast ‘cereals’, ‘cereal’ and ‘energy’ bars, ‘energy’ drinks, milk drinks, ‘fruit’ yoghurts and ‘fruit’ drinks, ‘cocoa’ drinks, ‘instant’ sauces; infant formulas, followon milks, ‘health’ and ‘slimming’ products (e.g. mealreplacement shakes and powders). Ready-to-heatmeals (e.g. pre-prepared pies, pasta and pizza), poultry and fish ‘nuggets’ and ‘sticks’, sausages, burgers, hot dogs, and other reconstituted meat products, powdered ‘instant’ soups, noodles and desserts.

Adapted from Monteiro et al., 2019, Juul et al., 2025, and Vadiveloo et al., 2025 [13], [19], [37]

The purpose and degree of processing are not interchangeable concepts; UPF are not simply industrial foods (as industrial processes span all Nova groups [13]), or foods with a high degree of physical processing. This distinction between purpose and degree of processing has typically not been acknowledged when comparing differences between classifications (akin to grouping different nutrient profiling models (NPM)) [12, 15]. Furthermore, food security gained from technological advances is not exclusive to UPF. MPF, processed culinary ingredients (PCI) and processed foods (PF) can all be fortified or contain functional additives to ensure food safety and durability [13]. The term ‘ultra-processing’ has also caused confusion regarding the role of formulation (the ingredients and additives used) [16–18]. The Nova classification intends to cover both processing and formulation, to capture how downstream food-level characteristics are shaped by upstream food systems drivers. For ‘ultra-processing’, these are processes and formulations enabling maximal profit (Table 1) [19]. Therefore, ‘ultra-profitable’ food, while not a term used in the original definition of the Nova classification, is perhaps a more convenient way to conceptually understand the purpose of UPF to maximise industry profits as per the purpose of processing aspect of the Nova classification [13, 19].

Given its originality, the Nova classification has been proposed to offer unique insights into diet and health [13, 19]. But, its use has also been questioned [20]. However, reviews either only discuss its value, and sometimes with overstated claims with potential unintended consequences [13, 19], or, reviews only discuss limitations [20, 21]. However, critical reviews discuss the degree, and not purpose, of processing [20, 21], discuss processing in general, and provide limited constructive commentary. Combined, this conflict and confusion overlooks genuine limitations of the Nova classification, and impedes progress to improve public health. Therefore, this review provides a balanced discussion of the purpose of processing and the Nova classification, with an overview of its potential and pitfalls largely overlooked to date, recommendations to avoid unintended consequences from prevailing narratives, and a constructive path forward that aligns perspectives.

## Potential

### The UPF Business model – the Ultra-Profitable Cycle

**Fig. 1:**
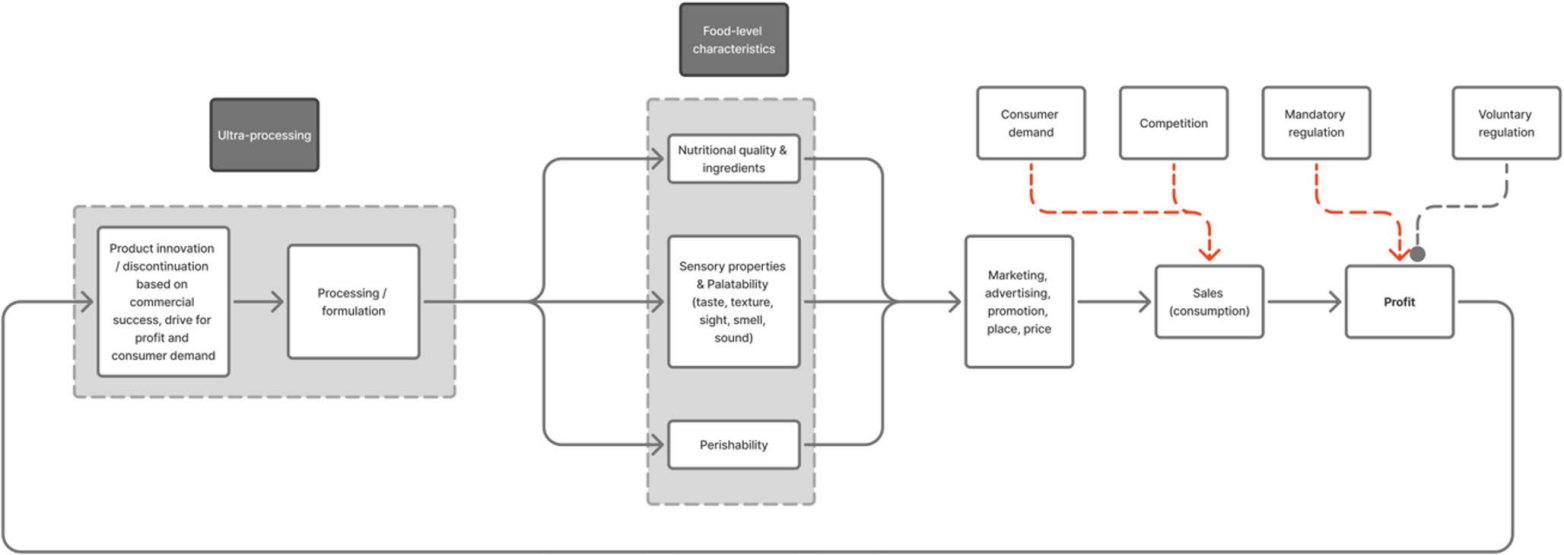
The ‘ultra-profitable’ food cycle: the financial drivers and reinforcing loops behind the creation and expansion of ultra-processed food. Adapted from [14], under an Open Government Licence CC 3.0. The purpose of processing is an upstream determinant that shapes food-level characteristics; the purpose determines the product design. In contrast, other food processing classifications focussing on the degree of physical processing assess foods based on food-level characteristics. Red dashed lines indicate external factors that impact on sales/profit. Grey dashed lines represent external factors that do not impact on sales/profit. ‘Ultra-profitable’ food, while not a term used in the original definition of the Nova classification, is perhaps a more convenient way to conceptually understand the purpose of UPF to maximise industry profits as per the purpose of processing aspect of the Nova classification.

The purpose of processing offers the potential to understand how upstream financial drivers and sociopolitical factors within the food system shape the downstream characteristics and availability of food, and the key actors involved (large multinational food corporations supplying most of the global energy supply) [3, 22]. Reductionist approaches to nutrition research do not capture how food systems shape food-level properties linked with health [23]. Thus, the Nova classification presents a conceptual shift away from viewing food-level characteristics and their accessibility as passive qualities, and towards considering the intention behind their existence. This has parallels to the ‘junk food cycle’ (Fig. 1) [14, 22].

### Product, Price, Place, Promotion

The unrelenting drive for commercial growth has developed into an ‘ultra-profitable’ feedback cycle. As population growth stabilised, routes for commercial growth could only be achieved through greater sales per capita. This requires creating more eating occasions, expanding product ranges, and/or displacing MPF [8, 22]. With no limit to financial success, but high market competition [8], corporations have continually battled for consumer attention and purchasing in a ‘survival of the fittest’ environment [8]. The product design and its price, promotion and placement (the 4Ps framework) are optimised to leverage the four broad drivers of food choice (availability, accessibility, affordability and desirability) for maximum profit [24]. Cheap industrial ingredients (widely available food substances of no or rare culinary use) are chosen to offer lower costs. Cosmetic additives are used to mask undesirable qualities resulting from using industrial food substances, or to enhance sensory properties to displace MPF or outcompete UPF competitors. To meet increasing sales and growth targets, these products are continually innovated and redesigned to optimise desirability and value across all sensory characteristics (e.g. sight and presentation, smell, taste, touch, shape, mouthfeel, and even sound) [25]. Every aspect is fine-tuned through extensive consumer sensory testing to find the most desirable qualities (e.g. ‘bliss point’) [26–28] that ensures repeated purchasing [25]. An implication of this business model is that nutrition and the environment are only considered through the lens of commercial success [29]. Products are only made ‘healthier’ for profit (e.g. to enter a new market or reformulate following mandatory regulation to avoid taxation). Likewise, commercially unsuccessful products, regardless of nutritional value, will be discontinued (Fig. 1). Thus, as a natural result of a purely financially-driven environment, there is an endless push to create the most appealing and profitable products that try to out compete each other, with a potentially never-ending range of new products [14].

Aggressive marketing tactics across the 4Ps are then used to drive sales [29]. Cheap ingredients allow the lowest price for the relevant market, extended product durability allows volume promotions (e.g. buy-one-get-one-free) and global distribution to make products highly accessible [29], and product packaging (e.g. colour, nutrition/health claims, promotional characters, pictured portion sizes, and messaging) is fine-tuned for maximum attractiveness [24]. Combined, these marketing tactics can negatively influence food preferences, purchases and eating behaviour, by shaping social norms around eating [14, 24, 30, 31]. This reinforces unhealthy habits and drives demand for and consumption of unhealthy food, which ultimately, strengthens the ‘ultra-profitable’ cycle [14, 24, 30, 31].

By implication, this cycle is closely linked with consumer behaviour. The cycle and 4Ps of UPF interact with consumer likes, wants and needs, which shapes the food environment and alters the development of products, such as choice of marketing strategies, and reformulation and discontinuation of healthier products to achieve sales growth. An example of the impact that this interaction can have on the cycle is from notable shifts in product development/reformulation resulting from changing consumer demands and choices from the rising use of glucagon-like peptide-1 receptor agonists.

The cycle also interacts with government and policymakers – where mandatory and voluntary regulations or policies can influence each aspect of the 4Ps – shaping product reformulation, pricing, promotion and placement (e.g. advertising restrictions, taxation, subsidisation, or food/additive safety authorisations).

### Capturing Broader Links Between Diet and Health

It is proposed that the recent rise in diet-related disease is linked with the rise in UPF [32]. Indeed, as defined by the Nova classification, available UPF and typical high-UPF diets are nutritionally poor and low in healthy food [33–35]. Furthermore, high-UPF diets are associated with increased risks of adverse health outcomes, including obesity, cardiometabolic disease and premature mortality [36]. Importantly, UPF may capture a broader picture of food characteristics linked with health than traditional nutrient- and food-based dietary guidance or policy [19, 37]. In cohort studies, nutrient intake or adherence to healthy dietary patterns do not explain the associations between UPF intake and poor health [38, 39]. In clinical trials in metabolic wards [40, 41] and free-living settings [42], nutritionally-matched *ad libitum* UPF versus non-UPF/MPF diets show significant differences in energy intake and weight change. Besides nutrients and foods, these wider UPF properties include sensory aspects that can be optimised by financial drivers that influence oro-sensory exposure time, eating rate, the potential for overconsumption and weight gain [8, 43–45], as well as influences on food wanting and reward [37, 39], appetite and craving control independent of diet quality [42], and the role of cosmetic additives [19].

### Confounder or Mediator?

There have been suggestions to control for properties such as texture, energy density, or the resulting energy intake rate when assessing the health impact of UPF [21, 46, 47]. These properties can be manipulated in food [20, 45, 46, 48, 49], and in principle, even reformulated to reduce energy intake [20, 46, 48–51]. However, in the ‘ultra-profitable’ cycle, financial drivers explicitly act upon the innate human preference for highly palatable and energy dense food when creating UPF [14]. Indeed, consumer sensory evaluation is fundamental to product development to optimise palatability and attractiveness to ensure product selection, and thus profit [26–28]. And, the biggest concern for manufacturers regarding reformulation is consumer acceptance and the risk that reformulation negatively impacts on enjoyment, which inadvertently motivates consumers to choose a competitor and therefore risk financial loss [52]. These characteristics do not need to be mutually exclusive (non-overlapping) across Nova groups for them to be optimised by financial drivers, but higher for UPF versus non-UPF across like-for-like comparisons. The optimisation of these characteristics in UPF is evident in the food supply [19, 37]. UPF are significantly more energy dense than MPF, and even nutritionally improved UPF are significantly more energy dense than comparable MPF [34]. In clinical trials, eating rate significantly increases from MPF, to PF, to UPF, with the eating rate of UPF nearly double that of MPF [46]. In clinical trials of nutritionally-matched, representative UPF/non-UPF diets over several weeks, the non-beverage energy density of UPF diets is higher than MPF diets [40–42]. In a single-day trial to determine the independent and combined effects of texture and (degree of) ultra-processing on energy intake, researchers were unable to control UPF and MPF meals for energy density, with the UPF diet being 0.20 kcal/g higher [49]. When energy density was matched in a single-meal trial assessing texture and/or processing level on energy intake, the ‘UPF’ meals were only 29% and 52% UPF, and nutrients (e.g. salt, and fibre) were not matched [48]. Textural changes are directly implicated in UPF from processing, formulation (multiple ingredients and cosmetic additives such as gelling agents or thickeners), and optimising sensory properties.

Suggestions to control for energy density, texture, and/or eating rate fails to acknowledge the upstream commercial incentives shaping these properties [21, 46, 47], and assumes their independence [46]. This also does not reflect the current food supply, and would constitute overadjustment (however, their control can help identify their relative importance as mechanisms of UPF). The financial drivers and reinforcing loops of the ‘ultra-profitable’ cycle act only to optimise these properties for consumer appeal, which prevents any self-sabotaging voluntary reformulation that impacts on sales (discussed in Path Forward) [29, 52]. And, even if consumer acceptance is not impacted, financial drivers of food production focus on food volume [14, 22]. Therefore, adjusting products to intentionally reduce consumption is financially counterproductive and in direct opposition to sales targets.

### Summary of Potential

By considering the purpose of processing, the Nova classification links how upstream economic and sociopolitical factors drive a reinforcing ‘ultra-profitable’ cycle that shapes downstream food characteristics and their availability. This provides a broader picture of diet-disease relationships that complements existing food-based dietary guidance and policy, and shifts the focus towards food systems as determinants of healthy diets.

## Pitfalls

However, there are also pitfalls to considering the purpose of processing using the Nova classification. If unaddressed, these pitfalls can have avoidable unintended consequences.

### Application of the Nova Classification

First, classifying food based on the purpose of processing must be operationalised [17]. However, the primary intent behind creating a product is not explicitly reported by food corporations, and must be inferred [13, 19]. Current approaches use objective coding based on the presence of markers of ultra-processing (industrial ingredients of no or rare culinary use or cosmetic additives) (Table 1), or subjective coding with thematic analysis, when objective approaches are not feasible [13, 19]. For detailed dietary tools such as food diaries or 24-hour recalls, product names, brands, food groups, and purchase locations can be thematically analysed to identify codes linked to the latent theme of the purpose of processing [53, 54]. For many items, ingredients lists on product labels or websites can be objectively reviewed for markers of ultra-processing [37]. However, for some reported foods or tools such as food frequency questionnaires (FFQ), less information is available [17]. This can impact on inferring the primary purpose of the products’ creation and confidence in coding. Less comprehensive tools such as FFQs may also limit the ability to distinguish between similar UPF vs. non-UPF foods (e.g. MPF vs. UPF lasagna). Thus, applying the Nova classification may pose pitfalls for coding agreement and accuracy in estimating UPF intake [39].

For many basic scientists, the subjectivity of thematic analyses presents a divergence away from conventional quantitative tools to analyse food, such as NPMs [55, 56]. This has led to some concerns and confusion around applying the Nova classification [39]. Whilst NPMs can often be readily applied, qualitative coding of ambiguous items in dietary tools requires training [53, 54]. Training must ensure a detailed understanding and familiarisation of the theme (Nova classification), data (dietary tool, and/or food and nutrient database being coded), and codes linking the two (e.g. product type, brands, markers). With training, there is good agreement across coders in identifying UPF [39], with many independent research teams applying the Nova classification subjectively and objectively [39]. Insufficient training therefore presents a barrier to correctly applying the Nova classification and potential for misclassification and confusion [39]. Table 2 provides an overview of best practice recommendations and resources to overcome potential pitfalls of misclassification or inaccuracy in operationalisation.

**Table 2:** Addressing potential pitfalls of applying the Nova classification, adapted from Dicken and Batterham, 2024 [39].

Domain	Recommendations
Training in applying the Nova classification	Ensure full training before performing coding. Follow published studies outlining the qualitative and quantitative coding processes for reproducibility. Follow best practice recommendations on the Nova classification, including for FFQs, 24-hour recalls, and decision flow charts.
New tools	Develop and validate Nova-specific tools to measure intake based on purpose of processing, for various settings (e.g. Nova-specific FFQ, screener, or 24-hour recall), and adapt existing tools to ask for information to guide inference of the purpose of processing (e.g. brands, product labels, markers). Utilise more advanced measurement tools and methods, such as metabolomic profiling of UPF, or AI-based approaches and machine learning to code based on themes. Develop and utilise detailed and updated food and nutrient databases through mandatory food corporation reporting on products, and sales, to directly link financial drivers and food-level properties (discussed in Path Forward).
Validation and Reproducibility	Continued validation of existing and novel tools to measure UPF intake, including reproducibility studies. To date, estimates of UPF intake have a similar level of reproducibility as estimates of energy-adjusted nutrient intake. Validation studies demonstrate a similar level of agreement between FFQs and 24-hour recalls or diet diaries in estimating UPF intake. Both FFQs and 24-hour recalls are directionally consistent in showing higher UPF intake is linked with poorer health, with larger effect sizes from 24-hour recalls than FFQs [80].
Analysis	Report the level of agreement in coding items according to the Nova classification by independent researchers, the extent of uncertainty in coding ambiguous items between researchers, the extent of training on coding, the coding approach used (e.g. subjective, objective), and the best practice recommendations that were followed. Perform sensitivity analyses for exposure-outcome associations using upper and lower estimates of UPF intake, based on alternative coding of UPF ambiguous items (i.e. changes in coding assumptions based on different starting assumptions of the intended purpose (e.g. [59])).

Abbreviations:* FFQ* food frequency questionnaire, UPF: ultra-processed food

### The Narrative of UPF as Intrinsically Unhealthy

**Fig. 2:**
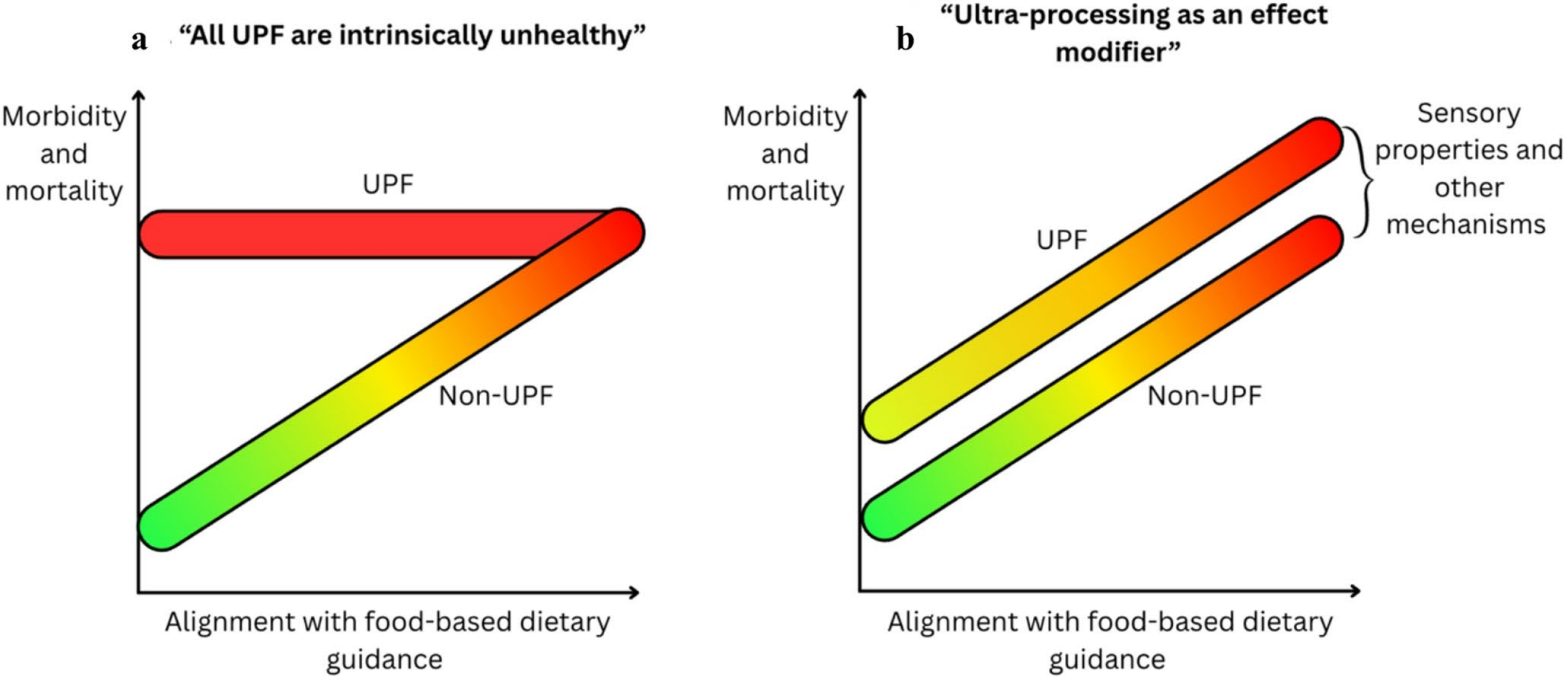
Potential framework of UPF as intrinsically unhealthy (**a**), or as an effect modifier of current healthy dietary guidance (**b**) [38–42]. a: conceptual view of ‘intrinsic unhealthy’ UPF and the health impact of UPF vs. non-UPF across diet quality according to current healthy dietary guidance; b: conceptual view of UPF as an effect modifier and health impact of UPF vs. non-UPF across diet quality according to current healthy dietary guidance. Abbreviations: UPF: ultra-processed food

UPF have been described as ‘intrinsically unhealthy’ [13] and not ‘real’ food [13], or it is claimed that processing, not food or nutrients, is what matters for health [57]. Whilst UPF may collectively reflect an unhealthy food system [22, 58], it is important to determine the direct biological health impacts of various types of UPF. These claims imply some underlying explicit act of harm by all food corporations in maximising profit. More realistically, financial incentives shaping product design inadvertently comes at the expense of food-level factors linked with poorer long-term health (e.g. nutritional quality, energy density, sensory properties). Furthermore, the health impact of different UPF varies in observational studies [59–61], with contrasting energy intake and weight change in *ad libitum* trials of UPF diets with varying characteristics [40–42, 62]. But, there is also a consistent effect of less favourable outcomes with high- versus low-UPF diets across both cohort studies and clinical trials [38–42, 63], even after accounting for diet quality (nutrients/diet pattern) [38–42]. Therefore, UPF are unlikely ‘inherently unhealthy’, and the impact of UPF on energy intake, weight and major health burdens will vary depending on the UPF studied. But, there appears to be an effect of ultra-processing in the overall diet, in addition to existing dietary guidance (Fig. 2).

The narrative of intrinsic harm may also relate to objectively identifying UPF using cosmetic additives or industrial ingredients of no or rare culinary use [13], making them seem the primary health concern per se [64]. For example, the UPF concept has been adopted by actors with ‘anti-sugar’ [65], ‘anti-seed oils’, ‘anti-artificial’ and ‘anti-synthetic chemicals’ agendas [64, 66]. These views may stem from the appeal to nature, that most cosmetic additives have no functional purpose [17, 67], or that many additives only recently but rapidly entered the food supply [68]. Even so, natural cosmetic additives (e.g. flavourings) still define UPF [69], and MPF, PCI and PF can be artificially fortified, or contain artificial additives for functional purposes (e.g. preservation [19]). And currently, most evidence linking additives with poor health is from lab studies [67, 70]. In limited human evidence, some specific additives may adversely impact mechanisms of disease pathways, including changes to gut microbiota and gut health [67, 70], inflammation [68, 70], and appetite regulation [71], with some positive associations with chronic disease risk [72, 73]. Several hundred additives are approved as safe for use by regulatory bodies, including the European Food Safety Authority and Food and Drug Administration [74]. Of which, many are consumed at levels naturally found within MPF (e.g. pectin), only a handful have evidence of possible harm at dosages consumed [37], and their presence varies across UPF. Thus, the evidence to date on cosmetic additives is insufficient to ascribe equal health harm to all UPF [70]. However, the role of additives in human disease requires further research, given that safety evaluations are based on acute toxicological assessments only, and not the cosmetic role of additives on changing sensory properties that influence eating behaviour, energy intake and long-term health. Therefore, a continued case-by-case re-evaluation of additives is needed, requiring improved research methods to generate stronger evidence of any harm (discussed in Path Forward).

### Pitfalls

Importantly, the narrative of intrinsically harmful UPF and focus on cosmetic additives and industrial/artificial ingredients impacts on the reporting and interpretation of UPF. This has knock on consequences, including impacting consumer perceptions of UPF, less effective product reformulation, less effective dietary substitutions, and downplaying reformulation strategies.

### Consumer Demand and Reformulation

First, media reporting on UPF shapes consumer perceptions, concerns, and demands [75–77]. This is important when media (and other public-facing communications such as consumer mobile apps to identify UPF), or influential actors with underlying agendas that deviate from the evidence [64–66], overemphasise UPF mechanisms with limited evidence (e.g. artificial ingredients of UPF linking with premature mortality) [78, 79]. Given the rapid rise in media attention, consumers are increasingly aware of, and concerned about, UPF [75–77]. Across Europe, 67% of consumers agree that UPF are harmful, and only 31% agree that UPF can be healthy [77]. Whilst the typically poor nutritional profile of UPF is noted by some consumers, 67% report not liking ingredients they do not recognise [77], with concerns about UPF being less ‘natural’ [76]. As such, consumers use ‘chemicals’, and artificial or unfamiliar ingredients to identify UPF [76, 77]. In the financially-driven ‘ultra-profitable’ cycle, consumer concerns can be used to design new products to drive sales, regardless of alignment with health impact (Fig. 1). For example, opting to remove cosmetic additives with no strong direct evidence of health harm from an energy-dense, nutritionally-poor UPF, or replacing an industrial ingredient for its non-industrial alternative with no change in nutritional value (e.g. swapping sugar for sugar; high-fructose corn syrup for honey). The result is less effective reformulation, but the marketing of a ‘healthier’ product.

### The Nova Classification and Dietary Guidance

This narrative may also suggest that replacing any UPF with non-UPF would lower health risks (Fig. 2a). However, the Nova classification is largely indifferent to dietary guidance, or food origin (plant vs. animal) [37, 80]. Total UPF avoidance when performed without consulting current dietary guidance or appropriate food substitutions may have unintended consequences from less effective dietary substitutions (e.g. increased salted butter or red meat consumption). Food and nutrients do matter (Fig. 2b). But, greater value may be obtained when dietary guidance and the Nova classification are combined [38, 39, 42]. The Nova classification compliments, not contradicts or replaces, food- and nutrient-based guidance [42].

### Focus on Mechanisms with Stronger Evidence and the Largest Health Burdens

**Fig. 3:**
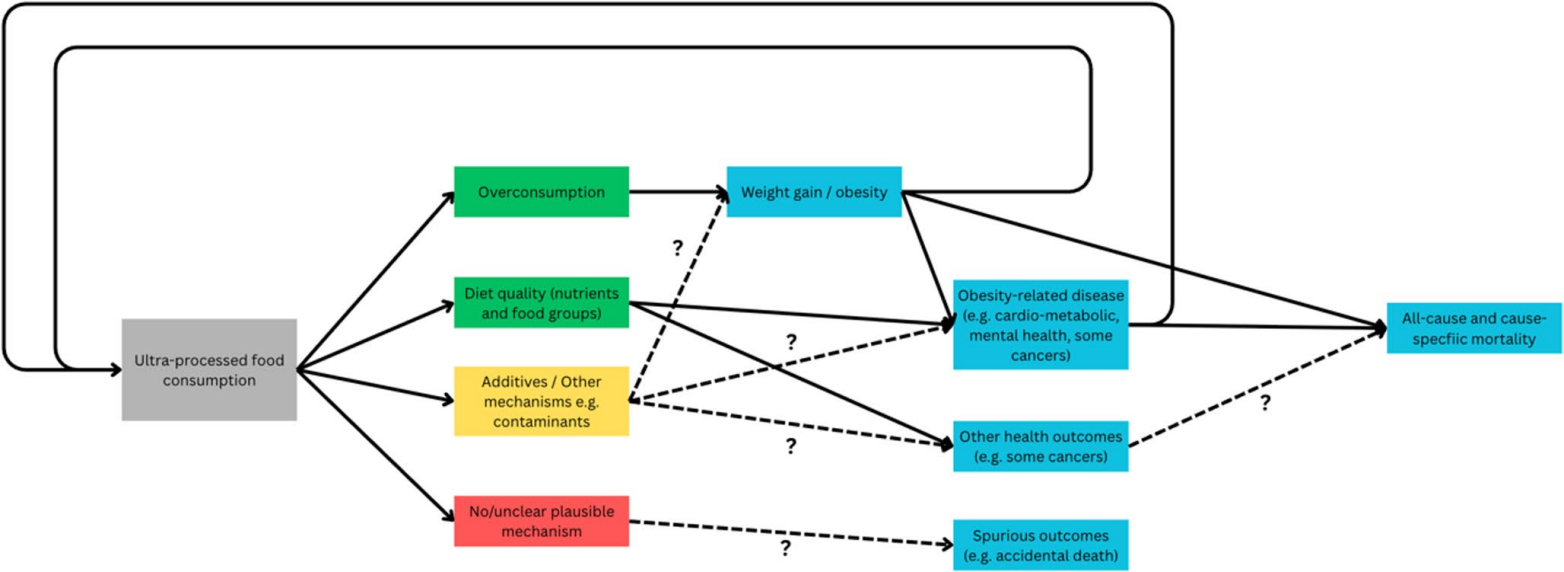
Diagram of relative strength of evidence between ultra-processed food intake, mechanisms and health outcomes. Green indicates a clear mechanism linking UPF with health outcomes with stronger evidence and larger effect size, amber indicates a potential mechanism linking UPF with health outcomes but with less clear evidence and less certain effect size, and red indicates no clear mechanism with outcomes associated with UPF intake. Dashed lines indicate mechanism-disease links that require further evidence to support a major role

A further pitfall of this narrative is that it also distracts from key UPF mechanisms with stronger evidence linked to greater health burdens, with proponents downplaying the potential benefit of addressing these properties as policy targets for reformulation or regulation [32, 52, 57]. Indeed, mounting evidence links UPF with poor health [36, 40–42], but the relative quality and strength of evidence varies across different mechanisms and outcomes (Fig. 3) [36, 39]. Stronger evidence links nutritional quality and sensory properties of UPF with overconsumption and unfavourable weight change [39–42, 49, 81], and UPF with obesity and obesity-related cardiometabolic disease [39], which are leading global health concerns [39]. In contrast, evidence linking UPF with other major conditions such as mental health [36], or rare conditions, are mostly cross-sectional [82], and the causal direction/relationship is unclear [82]. For example, there are bidirectional links between obesity and mental health [83], and some studies instead model mental health as the exposure, and UPF as the outcome [82]. Whilst causal relationships may exist, critical assessment of plausible mechanisms and evidence quality is necessary. For example, UPF has also been linked with accidental death, where there is no plausible mechanism [84]. Therefore, reporting and action on UPF should be prioritised based on the strength of evidence and size of effect of UPF mechanisms with major health burdens, with further research to better understand UPF-outcome associations with lower population burden and less evidence.

### UPF in Unique Cases

Lastly, UPF are used for medical purposes, as nutritional aids in humanitarian crises, and fuelling during extreme endurance sport. These specific cases are distinct to public health nutrition, and do not discount the wider evidence on UPF in public health. If deemed necessary, the role of UPF in such scenarios requiring specific nutrient or energy needs should undergo risk-benefit analysis and/or evaluation of non-UPF comparators in randomised controlled trials to determine effectiveness and safety. Importantly, the Nova classification highlights when these cases are exploited for financial gain with minimal health benefit, such as the growth of ‘sports nutrition’ products with health halos and nutrition claims, designed to displace MPF in the general diet [85].

In summary, pitfalls of the Nova classification include its practical operationalisation, narrative of being intrinsically unhealthy, overfocus on UPF mechanisms with weaker evidence, and its usage to inform diets in isolation, with potential inadvertent consequences on diet and policy. However, these can be avoided through adequate training on applying the Nova classification, continued improvement and validation of methods to identify UPF, improved communication on UPF by prioritising mechanisms based on strength of evidence and size of effect with major health burdens, and using the Nova classification alongside food-based dietary guidance.

## The Path Forward

### A new UPF Classification, or Improved Identification?

There have been calls for a new UPF classification [86]. However, it is important to distinguish between a new classification based on the purpose of processing (replacing the Nova classification), and a new operational approach (applying the Nova classification to identify UPF). New operational approaches can be valuable if they improve agreement in identifying UPF, understanding of mechanisms, and application of research findings. However, they must balance complexity with added value, and consider whether objective markers to identify UPF signifies direct causal mechanisms, or passive markers of causal mechanisms linked to the purpose of processing (thus risking misinterpretation as the direct causal effect, e.g. the role of cosmetic additives).

### A Systems Approach to Identifying UPF

Importantly, whilst there have been validation studies on the operational application of the Nova classification, there have been no validation studies assessing the link between the concept of the Nova classification, and its operational application (i.e. are the specified markers of ultra-processing a fair reflection of financial drivers shaping UPF). New operational approaches still implicitly assume the primary processing purpose [39]. A better approach therefore may be to explicitly identify the purpose of processing. Leading food corporations (including the out-of-home sector) can report on products, sales, profits, discontinuations and reformulations, to build a comprehensive product database containing food properties shaped by financial drivers (e.g. ingredients and quantities, nutrients, sensory properties, portion sizes). Ideally, this would also report the processes involved, but may be limited by intellectual property rights (Table 1). Connecting the database with dietary assessment tools facilitates higher-quality research that allows deducing which key UPF properties shaped by financial drivers have the greatest impact on health. For example, examining real-world exposures of additives and long-term health [67], [70], or alongside feeding trials [46], identifying products exceeding upper limits of properties (e.g. palatability, texture, energy density, energy intake rate and related cosmetic additives) that promote excess intake.

### Critical Arguments are Needed

Science should be open to limitations and improvement, and the Nova classification is no different. However, most appraisals do not address the purpose of processing [18], [20], [21], use strawman arguments, critique the degree, not purpose, of processing [18], [20], [21], [87], [88], debate food processing in general [18], [20], [88], use false dilemma [20], and arguments from analogy [20], [46]. The issues with such arguments were previously flagged [32], [89], yet are still used [18], [20], [21], [87], [88]. By not addressing the purpose of processing, this leads to confusion and misplaced concern regarding UPF according to the Nova classification. Likewise, claims that UPF are intrinsically unhealthy, not real food [13], or food nor nutrients matter for health [57], also impedes collective stakeholder agreement on effective policy. The UPF term is now widely used in the literature, media and by the public, and likely here to stay. Therefore, there is a need for appropriate communication on UPF, which may differ for researchers, policy makers and the public, particularly clarifying whether it is defined by the Nova classification or not.

### Improving the Food System

Moving forwards, stakeholders must focus where evidence strongly aligns across all scientific disciplines. There is widespread agreement amongst academics [10], [90], governments [90], [91], non-governmental organisations [92], [93], and senior politicians [94], that the current food system is a key driver of diet-related poor health [1]. As a natural consequence of the potential for unlimited financial growth at the expense of health and the environment, the transformation in the food system that has provided security for billions has now itself become a barrier for billions more to access and afford healthy food [1], [9], [14]. Importantly, the most deprived are worst impacted [9], [14], [90], with diet-related disease being strongly linked with socioeconomic status and deprivation level [14], [90]. Furthermore, in global regions of high agricultural productivity, individuals still suffer from food insecurity and malnutrition as a result of commercial and political drivers exporting crops for profit [95].

### Action Needed Should be Higher Level Issue with Current Policy

There is agreement that improving access to and affordability of healthy food requires higher-level, food systems transformation [90], [91], [92], [93]. However, most current regulatory responses fall on individual responsibility [4], [5], [22], [90], [96]. These require high levels of individual agency, and are less equitable and effective than lower-agency, population-level actions (e.g. subsidising healthy food or mandatory reformulation), which instead widens health inequalities [96]. Lower agency interventions are often seen as unfavourable by governments, potentially from political ideology (arguments of a ‘nanny state’, despite the lack of choice in affordability and accessibility in the current food environment) or competing interests with industry [94], and by commercially-invested food corporations [96]. The financial interests of the commercial food industry are misaligned with public health interests [29], [97], who oppose health-related proposals that restrict their products with multiple strategies (e.g. disputing the health benefits of proposals with unsubstantiated or misleading claims, or by lobbying) [29], [90], [94]. Governments and stakeholders must be aware of industry influence that prevents food systems change to improving access to healthy food [90], [94], [96].

### Complexity Required to Change and Include Health as a Stakeholder

There are currently no incentives for the commercial food industry to provide affordable healthy food [14], [29]. Commercial success can be achieved within a healthy and sustainable food system [14], [29], but fundamental changes are needed to move from unrestricted economic growth as the sole driver of the food system, to economic growth, health and the environment [22], [29]. This transformation requires significant shifts in the governance and fundamental workings of the food system [98], particularly by governments [29], and government-led extra-governmental bodies, who are held accountable by wider stakeholders [98]. Academics, policymakers and the commercial food industry must communicate to enact change, requiring clearly defined working relationships.

### Corporate Transparency

Major food corporations need to be held accountable, providing transparency through mandatory reporting on products and sales [90]. Food databases containing products, characteristics and sales facilitates improved research to determine links between commercially shaped food characteristics and poor health. This represents a key step by the commercial food industry in working with government to support policymakers in effective policy design, and creating a healthier food environment [90].

No policy alone will improve access to healthy food, and all strategies should be utilised alongside whole-food reformulation [97], particularly for countries with limited public health resources or widespread availability of UPF. Importantly, current food-based dietary guidance and policies do not capture the full extent of diet-disease relationships [38], [42]. Several food properties shaped by financial drivers and implicated in UPF remain unaddressed in policy, despite their influence on energy intake and weight management [24], [30], [44], [50], [81]. These could be directly addressed in policy or dietary guidance without explicitly using the Nova classification (e.g. synergistic regulation across the 4Ps; setting regulatory thresholds on sensory properties promoting excess intake). Moving forwards, both formulation and processing must be considered to comprehensively address product characteristics influencing health. Current nutrients-to-limit reformulations alter single aspects, which, despite being heavily marketed with health or nutrition claims, only marginally improves nutrient quality [99]. Furthermore, there may be unintended impacts of health or nutrition claims on eating behaviour, such as underestimating energy content and inadvertently increasing portion sizes [100]. Importantly, reformulation comes at a cost to corporations [52], and as health-concerned consumers will pay more for healthier products [101], reformulations can be priced and marketed at a premium. Unless reformulations replace the original product as an affordable and accessible option, there will be minimal public health improvement, and will inadvertently widen health inequalities by outpricing the most disadvantaged. Corporations will not invest in costly reformulation that risks compromising profit when there is no mandate to do so [52]. Effective reformulation therefore requires incentivisation or mandatory regulation to ensure change and equitable access. This is evident by the greater effectiveness of mandatory policies to improve food, following ineffective self-regulatory/voluntary approaches [90], [102], [103].

### Investment

Significant social, financial and intellectual investment is required to attain the long-term benefits of improved access to healthy diets from these systems-level structural changes. This will include social investment to prioritise public health and sustainability, and build cross-governmental organisations free of industry influence and balanced power dynamics, financial investment for multi-faceted mandatory policies that prioritise public health and sustainability, and multi-disciplinary research spanning food systems and biology, and intellectual investment to build comprehensive real-time product databases for research and policy enforcement, and where needed to promote action, realising to policymakers the long-term value of higher-level food systems changes to support public health and sustainability.

In summary, there is wide agreement on transforming the food system to incentivise the production, availability and accessibility of healthy food. A path forward that focusses on health and the environment alongside profit can benefit from considering the purpose of processing, without necessarily explicitly using the Nova classification in policy.

## Conclusions

Food systems have become increasingly globalised from commercial incentives to provide a sufficient energy supply. This has inadvertently led to a food system that makes accessing and affording healthy food difficult for billions worldwide. By considering food based on the purpose of its creation, the Nova classification offers a framework linking upstream economic and sociopolitical factors with downstream properties of available food, complementing existing food-based dietary guidance. However, its practical application, narrative of intrinsic harm of UPF, usage to guide diets in isolation, and overemphasis on mechanisms with weaker evidence are pitfalls, that whilst avoidable, can have unintended consequences. There is wide agreement on the need for food systems transformation to ensure access and affordability to healthy diets for all. For public health, the purpose behind food processing provides an understanding of systems drivers of food production. This can be reflected in policy to improve access to healthy diets, without necessarily explicit use of the Nova classification.

## Key References

- F. Juul, E. Martinez-Steele, N. Parekh, and C. A. Monteiro, ‘The role of ultra-processed food in obesity’, Nat Rev Endocrinol, pp. 1–14, Jul. 2025. 10.1038/s41574-025-01143-7.
  - **○** This review article provides a provides a comprehensive background explaining the rise in the ultra-processed food system, and detailed discussion on the evidence linking ultra-processed food intake as defined by the Nova classification with obesity, including wider discussion on future research considerations.
- J. Adams, ‘The NOVA system can be used to address harmful foods and harmful food systems’, PLOS Medicine, vol. 21, no. 11, p. e1004492, Nov. 2024. 10.1371/journal.pmed.1004492
  - **○ **This article discusses the role of the Nova classification in helping to address the food system to improve access to healthy diets, and considers the potentially contrasting views between life scientists and social scientists in evaluating the Nova classification.
- S. J. Dicken et al., ‘Ultraprocessed or minimally processed diets following healthy dietary guidelines on weight and cardiometabolic health: a randomized, crossover trial’, Nat Med, pp. 1–12, Aug. 2025. 10.1038/s41591-025-03842-0
  - **○ **This article reports the first free-living randomised controlled feeding trial comparing UPF with MPF on weight and health, and also the first trial to do so in the context of healthy dietary guidance.

## Data Availability

No original data was accessed in this manuscript.

## References

[1] Afshin A et al. May., ‘Health effects of dietary risks in 195 countries, 1990–2017: a systematic analysis for the Global Burden of Disease Study 2017’, *The Lancet*, vol. 393, no. 10184, pp. 1958–1972, 2019, 10.1016/S0140-6736(19)30041-8 10.1016/S0140-6736(19)30041-8 PMC6899507 30954305

[2] FAO, IFAD, UNICEF, WFP, and WHO, The State of Food Security and Nutrition in the World 2025. FAO, IFAD ; UNICEF ; WFP ; WHO. ; ;, 2025. Accessed: Aug. 19, 2025. [Online]. Available: https://openknowledge.fao.org/handle/20.500.14283/cd6008en

[3] Baker P, et al. Ultra-processed foods and the nutrition transition: Global, regional and national trends, food systems transformations and political economy drivers. Obes Rev. 2020;21(12):e13126. 10.1111/obr.13126. 32761763 10.1111/obr.13126

[4] Theis DR, White M. ‘Is Obesity Policy in England Fit for Purpose? Analysis of Government Strategies and Policies, 1992–2020’, *The Milbank Quarterly*, vol. 99, no. 1, pp. 126–170, 2021, 10.1111/1468-0009.12498 10.1111/1468-0009.12498 PMC7984668 33464689

[5] Northcott T, Lawrence M, Parker C, Reeve B, Baker P. ‘Regulatory responses to ultra-processed foods are skewed towards behaviour change and not food system transformation’, *Nat Food*, vol. 6, no. 3, pp. 273–282, Mar. 2025, 10.1038/s43016-024-01101-y 10.1038/s43016-024-01101-y 39794395

[6] Forde CG, Decker EA. The Importance of Food Processing and Eating Behavior in Promoting Healthy and Sustainable Diets. Annu Rev Nutr. 2022;42(1):377–99. 10.1146/annurev-nutr-062220-030123. 35671530 10.1146/annurev-nutr-062220-030123

[7] van Boekel M, et al. A review on the beneficial aspects of food processing. Mol Nutr Food Res. 2010;54(9):1215–47. 10.1002/mnfr.200900608. 20725924 10.1002/mnfr.200900608

[8] Hall KD. From dearth to excess: the rise of obesity in an ultra-processed food system. Philos Trans R Soc Lond B Biol Sci. Jul. 2023;378(1885):20220214. 10.1098/rstb.2022.0214. 10.1098/rstb.2022.0214 PMC10363698 37482782

[9] Ambikapathi R, Schneider KR, Davis B, Herrero M, Winters P, Fanzo JC. ‘Global food systems transitions have enabled affordable diets but had less favourable outcomes for nutrition, environmental health, inclusion and equity’, *Nat Food*, vol. 3, no. 9, pp. 764–779, Sep. 2022, 10.1038/s43016-022-00588-7 10.1038/s43016-022-00588-7 37118149

[10] Dicken SJ, Batterham RL. Ultra-processed food: a global problem requiring a global solution. Lancet Diabetes Endocrinol. Oct. 2022;10(10):691–4. 10.1016/S2213-8587(22)00248-0. 10.1016/S2213-8587(22)00248-0 36037821

[11] Popkin BM, Ng SW. The nutrition transition to a stage of high obesity and noncommunicable disease prevalence dominated by ultra-processed foods is not inevitable. Obes Rev. 2022;23(1):e13366. 10.1111/obr.13366. 34632692 10.1111/obr.13366 PMC8639733

[12] Sadler CR, Grassby T, Hart K, Raats M, Sokolović M, Timotijevic L. Processed food classification: Conceptualisation and challenges. Trends Food Sci Technol. Jun. 2021;112:149–62. 10.1016/j.tifs.2021.02.059.

[13] Monteiro CA et al. Apr., ‘Ultra-processed foods: what they are and how to identify them’, *Public Health Nutr*, vol. 22, no. 5, pp. 936–941, 2019, 10.1017/S1368980018003762 10.1017/S1368980018003762 PMC10260459 30744710

[14] ‘The National Food Strategy - The Plan’. National Food Strategy. Accessed: Aug. 20, 2025. [Online]. Available: https://www.nationalfoodstrategy.org/

[15] Medin AC, Gulowsen SR, Groufh-Jacobsen S, Berget I, Grini IS, Varela P. Definitions of ultra-processed foods beyond NOVA: a systematic review and evaluation. Food Nutr Res. 2025;69. 10.29219/fnr.v69.12217. 10.29219/fnr.v69.12217 PMC12255158 40655201

[16] Botelho R, Araújo W, Pineli L. ‘Food formulation and not processing level: Conceptual divergences between public health and food science and technology sectors’, *Crit Rev Food Sci Nutr*, vol. 58, no. 4, pp. 639–650, Mar. 2018, 10.1080/10408398.2016.1209159 10.1080/10408398.2016.1209159 27439065

[17] O’Connor LE, Herrick KA, Papier K. Handle with care: challenges associated with ultra-processed foods research. Int J Epidemiol. Aug. 2024;53(5):dyae106. 10.1093/ije/dyae106. 10.1093/ije/dyae106 PMC11349190 39191478

[18] Levine AS, Ubbink J. Ultra-processed foods: Processing versus formulation. Obes Sci Pract. Aug. 2023;9(4):435–9. 10.1002/osp4.657. 10.1002/osp4.657 PMC10399516 37546281

[19] Juul F, Martinez-Steele E, Parekh N, Monteiro CA. The role of ultra-processed food in obesity. Nat Rev Endocrinol. Jul. 2025;1–14. 10.1038/s41574-025-01143-7. 10.1038/s41574-025-01143-7 40659796

[20] Forde CG. ‘Beyond ultra-processed: considering the future role of food processing in human health’, *Proc Nutr Soc*, vol. 82, no. 3, pp. 406–418, Sep. 2023, 10.1017/S0029665123003014 10.1017/S0029665123003014 37654079

[21] Gibney MJ, Forde CG. ‘Nutrition research challenges for processed food and health’, *Nat Food*, vol. 3, no. 2, pp. 104–109, Feb. 2022, 10.1038/s43016-021-00457-9 10.1038/s43016-021-00457-9 37117956

[22] White M. ‘Challenges for regulatory responses to ultra-processed foods’, *Nat Food*, vol. 6, no. 3, pp. 230–231, Mar. 2025, 10.1038/s43016-025-01138-7 10.1038/s43016-025-01138-7 40038528

[23] Dangour AD, Mace G, Shankar B. Food systems, nutrition, health and the environment. Lancet Planet Health. Apr. 2017;1(1):e8–9. 10.1016/S2542-5196(17)30004-9. 10.1016/S2542-5196(17)30004-9 29851593

[24] Boyland E et al. Mar., ‘Food marketing, eating and health outcomes in children and adults: a systematic review and meta-analysis’, *British Journal of Nutrition*, vol. 133, no. 6, pp. 781–805, 2025, 10.1017/S0007114524000102 10.1017/S0007114524000102 PMC12169957 40518855

[25] Rao P, Rodriguez RL, Shoemaker SP. Addressing the sugar, salt, and fat issue the science of food way. NPJ Sci Food. Jul. 2018;2(12). 10.1038/s41538-018-0020-x. 10.1038/s41538-018-0020-x PMC6550161 31304262

[26] Durrant LR, et al. Assessing the dietary intake of sensory panellists: implications for workplace nutrition and health. Int J Food Sci Tech. Jul. 2025;60:vvaf048. 10.1093/ijfood/vvaf048. no. 2.

[27] O’Sullivan M. A Handbook for Sensory and Consumer-Driven New Product Development: Innovative Technologies for the Food and Beverage Industry. Woodhead Publishing; 2016.

[28] Świąder K, Marczewska M. ‘Trends of Using Sensory Evaluation in New Product Development in the Food Industry in Countries That Belong to the EIT Regional Innovation Scheme’, *Foods*, vol. 10, no. 2, p. 446, Feb. 2021, 10.3390/foods10020446 10.3390/foods10020446 PMC7922510 33670555

[29] White M, Aguirre E, Finegood DT, Holmes C, Sacks G, Smith R. ‘What role should the commercial food system play in promoting health through better diet?’, Mar. 2020, 10.1136/bmj.m545 10.1136/bmj.m545 PMC7190369 32184211

[30] Boyland E, et al. Impact of food, beverage, and alcohol brand marketing on consumptive behaviors and health in children and adults: A systematic review and meta-analysis. Obes Rev. Sep. 2025;26(9):e13932. 10.1111/obr.13932. 10.1111/obr.13932 PMC12318912 40228497

[31] Boyland E. Would Reducing Children’s Exposure to Food Advertising Prevent Unhealthy Weight Gain? Curr Obes Rep. Jun. 2025;14(1):55. 10.1007/s13679-025-00648-6. 10.1007/s13679-025-00648-6 PMC12182458 40542916

[32] Monteiro CA, Cannon G, Moubarac J-C, Levy RB, Louzada MLC, Jaime PC. ‘The UN Decade of Nutrition, the NOVA food classification and the trouble with ultra-processing’, *Public Health Nutr*, vol. 21, no. 1, pp. 5–17, Jan. 2018, 10.1017/S1368980017000234 10.1017/S1368980017000234 PMC10261019 28322183

[33] Martini D, Godos J, Bonaccio M, Vitaglione P, Grosso G. ‘Ultra-Processed Foods and Nutritional Dietary Profile: A Meta-Analysis of Nationally Representative Samples’, *Nutrients*, vol. 13, no. 10, Art. no. 10, Oct. 2021, 10.3390/nu13103390 10.3390/nu13103390 PMC8538030 34684391

[34] Dicken SJ, Batterham RL, Brown A. Nutrients or processing? An analysis of food and drink items from the UK National Diet and Nutrition Survey based on nutrient content, the NOVA classification, and front of package traffic light labelling. Br J Nutr. 2024;131(9):1619–32. 10.1017/S0007114524000096. 38220223 10.1017/S0007114524000096 PMC11043912

[35] Dicken SJ, Batterham RL, Brown A. ‘Micronutrients or processing? An analysis of food and drink items from the UK National Diet and Nutrition Survey based on micronutrient content, the Nova classification and front-of-package traffic light labelling’, *Br J Nutr*, vol. 133, no. 3, pp. 1–18, Jan. 2025, 10.1017/S0007114524003374 10.1017/S0007114524003374 PMC11946030 39801244

[36] Lane MM et al. Feb., ‘Ultra-processed food exposure and adverse health outcomes: umbrella review of epidemiological meta-analyses’, *BMJ*, vol. 384, p. e077310, 2024, 10.1136/bmj-2023-077310 10.1136/bmj-2023-077310 PMC10899807 38418082

[37] Vadiveloo MK et al. ‘Ultraprocessed Foods and Their Association With Cardiometabolic Health: Evidence, Gaps, and Opportunities: A Science Advisory From the American Heart Association’, *Circulation*, vol. 0, no. 0. 10.1161/CIR.0000000000001365 10.1161/CIR.0000000000001365 40776885

[38] Dicken SJ, Batterham RL. ‘The Role of Diet Quality in Mediating the Association between Ultra-Processed Food Intake, Obesity and Health-Related Outcomes: A Review of Prospective Cohort Studies’, *Nutrients*, vol. 14, no. 1, Art. no. 1, Jan. 2022, 10.3390/nu14010023 10.3390/nu14010023 PMC8747015 35010898

[39] Dicken SJ, Batterham RL. Ultra-processed Food and Obesity: What Is the Evidence? Curr Nutr Rep. Jan. 2024;13(1):23–38. 10.1007/s13668-024-00517-z. 10.1007/s13668-024-00517-z PMC10924027 38294671

[40] Hamano S et al. Nov., ‘Ultra-processed foods cause weight gain and increased energy intake associated with reduced chewing frequency: A randomized, open-label, crossover study’, *Diabetes Obes Metab*, vol. 26, no. 11, pp. 5431–5443, 2024, 10.1111/dom.15922 10.1111/dom.15922 39267249

[41] Hall KD, et al. Ultra-Processed Diets Cause Excess Calorie Intake and Weight Gain: An Inpatient Randomized Controlled Trial of Ad Libitum Food Intake. Cell Metab. Jul. 2019;30(1):67–77. 10.1016/j.cmet.2019.05.008. 10.1016/j.cmet.2019.05.008 PMC7946062 31105044

[42] Dicken SJ, et al. Ultraprocessed or minimally processed diets following healthy dietary guidelines on weight and cardiometabolic health: a randomized, crossover trial. Nat Med. Aug. 2025;1–12. 10.1038/s41591-025-03842-0. 10.1038/s41591-025-03842-0 PMC12532614 40760353

[43] de Graaf C. ‘Texture and satiation: the role of oro-sensory exposure time’, *Physiol Behav*, vol. 107, no. 4, pp. 496–501, Nov. 2012, 10.1016/j.physbeh.2012.05.008 10.1016/j.physbeh.2012.05.008 22609070

[44] de Graaf C, Kok FJ. Slow food, fast food and the control of food intake. Nat Rev Endocrinol. May 2010;6(5):290–3. 10.1038/nrendo.2010.41. 10.1038/nrendo.2010.41 20351697

[45] Rolls BJ. ‘The relationship between dietary energy density and energy intake’, *Physiology & Behavior*, vol. 97, no. 5, pp. 609–615, Jul. 2009, 10.1016/j.physbeh.2009.03.011 10.1016/j.physbeh.2009.03.011 PMC4182946 19303887

[46] Forde CG, Mars M, de Graaf K. Ultra-Processing or Oral Processing? A Role for Energy Density and Eating Rate in Moderating Energy Intake from Processed Foods. Curr Dev Nutr. Mar. 2020;4(3):nzaa019. 10.1093/cdn/nzaa019. 10.1093/cdn/nzaa019 PMC7042610 32110771

[47] Astrup A, Monteiro CA. ‘Does the concept of ultra-processed foods help inform dietary guidelines, beyond conventional classification systems? NO’, *Am J Clin Nutr*, vol. 116, no. 6, p. nqac123, Jun. 2022, 10.1093/ajcn/nqac123 10.1093/ajcn/nqac123 35670128

[48] Lasschuijt M, Camps G, Mars M, Siebelink E, de Graaf K, Bolhuis D. ‘Speed limits: the effects of industrial food processing and food texture on daily energy intake and eating behaviour in healthy adults’, *Eur J Nutr*, vol. 62, no. 7, pp. 2949–2962, Oct. 2023, 10.1007/s00394-023-03202-z 10.1007/s00394-023-03202-z PMC10469122 37452167

[49] Teo PS et al. Mar., ‘Texture-based differences in eating rate influence energy intake for minimally processed and ultra-processed meals’, *Am J Clin Nutr*, vol. 116, no. 1, pp. 244–254, 2022, 10.1093/ajcn/nqac068 10.1093/ajcn/nqac068 PMC9257473 35285882

[50] Rolls BJ. Dietary energy density: Applying behavioural science to weight management. Nutr Bull. Sep. 2017;42(3):pp246–253. 10.1111/nbu.12280. 10.1111/nbu.12280 PMC5687574 29151813

[51] Bolhuis DP, Forde CG. ‘Application of food texture to moderate oral processing behaviors and energy intake’, *Trends Food Sci Technol*, vol. 106, pp. 445–456, Dec. 2020, 10.1016/j.tifs.2020.10.021

[52] Fanzo J, McLaren R, Bellows A, Carducci B. Challenges and opportunities for increasing the effectiveness of food reformulation and fortification to improve dietary and nutrition outcomes. Food Policy. Aug. 2023;119:102515. 10.1016/j.foodpol.2023.102515.

[53] Braun V, Clarke V. ‘Toward good practice in thematic analysis: Avoiding common problems and be(com)ing a knowing researcher’. Int J Transgend Health, 24, 1, pp. 1–6, 10.1080/26895269.2022.2129597 10.1080/26895269.2022.2129597 PMC9879167 36713144

[54] Braun V, Clarke V. ‘Using thematic analysis in psychology’, *Qualitative Research in Psychology*, vol. 3, no. 2, pp. 77–101, Jan. 2006, 10.1191/1478088706qp063oa

[55] Food Standards Agency and Department of Health. ‘Guide to creating a front of pack (FoP) nutrition label for pre-packed products sold through retail outlets’, Food Standards Agency. Accessed: Oct. 16, 2021. [Online]. Available: https://www.food.gov.uk/sites/default/files/media/document/fop-guidance_0.pdf

[56] Department of Health. ‘Nutrient Profiling Technical Guidance’, Department of Health, 2011. Accessed: Dec. 18, 2023. [Online]. Available: https://assets.publishing.service.gov.uk/government/uploads/system/uploads/attachment_data/file/216094/dh_123492.pdf

[57] Monteiro CA. Nutrition and health. The issue is not food, nor nutrients, so much as processing. Public Health Nutr. May 2009;12(5):729–31. 10.1017/S1368980009005291. 10.1017/S1368980009005291 19366466

[58] Adams J. The NOVA system can be used to address harmful foods and harmful food systems. PLoS Med. Nov. 2024;21(11):e1004492. 10.1371/journal.pmed.1004492. 10.1371/journal.pmed.1004492 PMC11575809 39561114

[59] Dicken SJ et al. Sep., ‘Food consumption by degree of food processing and risk of type 2 diabetes mellitus: a prospective cohort analysis of the European Prospective Investigation into Cancer and Nutrition (EPIC)’, *The Lancet Regional Health – Europe*, vol. 0, no. 0, 2024, 10.1016/j.lanepe.2024.101043 10.1016/j.lanepe.2024.101043 PMC11551512 39529810

[60] Dicken SJ. Are all ultra-processed foods bad for health? – Author’s reply. Lancet Reg Health Eur. Oct. 2024;46:101108. 10.1016/j.lanepe.2024.101108. 10.1016/j.lanepe.2024.101108 PMC11530753 39493248

[61] Vadiveloo MK, Gardner CD. ‘Not All Ultra-Processed Foods Are Created Equal: A Case for Advancing Research and Policy That Balances Health and Nutrition Security’, *Diabetes Care*, vol. 46, no. 7, pp. 1327–1329, Jun. 2023, 10.2337/dci23-0018 10.2337/dci23-0018 37339348

[62] Hägele FA, et al. Short-term effects of high-protein, lower-carbohydrate ultra-processed foods on human energy balance. Nat Metab. Apr. 2025;7(4):704–13. 10.1038/s42255-025-01247-4. 10.1038/s42255-025-01247-4 PMC12021659 40082711

[63] Preston JM, et al. Effect of ultra-processed food consumption on male reproductive and metabolic health. Cell Metabol. Aug. 2025;0(0). 10.1016/j.cmet.2025.08.004. 10.1016/j.cmet.2025.08.004 40882621

[64] HHS, Report’ ‘TheMAHA. The White House. Accessed: Aug. 21, 2025. [Online]. Available: https://www.whitehouse.gov/maha/

[65] Lustig RH. ‘Ultraprocessed Food: Addictive, Toxic, and Ready for Regulation’, *Nutrients*, vol. 12, no. 11, p. 3401, Nov. 2020, 10.3390/nu12113401 10.3390/nu12113401 PMC7694501 33167515

[66] Scherger J. ‘Dark Calories: How Vegetable Oils Destroy Our Health and How We Can Get It Back’, *Family Medicine*, vol. 57, no. 4, pp. 314–315, 2025, 10.22454/FamMed.2025.991804

[67] Seto T, Grondin JA, Khan WI. Food Additives: Emerging Detrimental Roles on Gut Health. FASEB J. 2025;39(13):e70810. 10.1096/fj.202500737R. 40622070 10.1096/fj.202500737R PMC12232514

[68] Partridge D, Lloyd KA, Rhodes JM, Walker AW, Johnstone AM, Campbell BJ. ‘Food additives: Assessing the impact of exposure to permitted emulsifiers on bowel and metabolic health – introducing the FADiets study’, *Nutr Bull*, vol. 44, no. 4, pp. 329–349, Dec. 2019, 10.1111/nbu.12408 10.1111/nbu.12408 PMC6899614 31866761

[69] Hess JM et al. Jun., ‘Dietary guidelines meet NOVA: developing a menu for a healthy dietary pattern using ultra-processed foods’, *J Nutr*, vol. 153, no. 8, pp. 2472–2481, 2023, 10.1016/j.tjnut.2023.06.028 10.1016/j.tjnut.2023.06.028 37356502

[70] Whelan K, Bancil AS, Lindsay JO, Chassaing B. ‘Ultra-processed foods and food additives in gut health and disease’, *Nat Rev Gastroenterol Hepatol*, vol. 21, no. 6, pp. 406–427, Jun. 2024, 10.1038/s41575-024-00893-5 10.1038/s41575-024-00893-5 38388570

[71] Chakravartti SP et al. Mar., ‘Non-caloric sweetener effects on brain appetite regulation in individuals across varying body weights’, *Nat Metab*, vol. 7, no. 3, pp. 574–585, 2025, 10.1038/s42255-025-01227-8 10.1038/s42255-025-01227-8 40140714

[72] Debras C, et al. Artificial sweeteners and cancer risk: Results from the NutriNet-Santé population-based cohort study. PLoS Med. Mar. 2022;19(3):e1003950. 10.1371/journal.pmed.1003950. 10.1371/journal.pmed.1003950 PMC8946744 35324894

[73] Debras C et al. Sep., ‘Artificial sweeteners and risk of cardiovascular diseases: results from the prospective NutriNet-Santé cohort’, *BMJ*, vol. 378, p. e071204, 2022, 10.1136/bmj-2022-071204 10.1136/bmj-2022-071204 PMC9449855 36638072

[74] European Food Safety Authority. ‘Food additives’, European Food Safety Authority (EFSA). Accessed: Jan. 19, 2024. [Online]. Available: https://www.efsa.europa.eu/en/topics/topic/food-additives

[75] Robinson E, Cummings JR, Gough T, Jones A, Evans R. ‘Consumer Awareness, Perceptions and Avoidance of Ultra-Processed Foods: A Study of UK Adults in 2024’, *Foods*, vol. 13, no. 15, p. 2317, Jul. 2024, 10.3390/foods13152317 10.3390/foods13152317 PMC11311829 39123509

[76] ‘Executive Summary | Advisory Committee for Social Science’. Accessed: Aug. 21, 2025. [Online]. Available: https://acss.food.gov.uk/Executive%20Summary

[77] ‘Consumer perceptions unwrapped: ultra-processed foods - EIT Food’. Accessed: Aug. 21, 2025. [Online]. Available: https://www.eitfood.eu/consumer-perceptions-unwrapped-ultra-processed-foods

[78] Campbell D. and D. C. H. policy editor, ‘Ultra-processed food increases risk of early death, international study finds’, *The Guardian*, Apr. 28, 2025. Accessed: Aug. 21, 2025. [Online]. Available: https://www.theguardian.com/society/2025/apr/28/ultra-processed-food-increases-risk-of-early-death-international-study-finds

[79] P. R. reporter, ‘Ultra-processed foods may be linked to early death’, BBC News. Accessed: Aug. 21, 2025. [Online]. Available: https://www.bbc.com/news/articles/crm30kwvv17o

[80] Mendoza K, Tobias DK. Quantity and Quality of Evidence Are Sufficient: Prevalent Features of Ultraprocessed Diets Are Deleterious for Health. Adv Nutr. Dec. 2023;15(1):100157. 10.1016/j.advnut.2023.100157. 10.1016/j.advnut.2023.100157 PMC10831941 38245357

[81] Fazzino TL, Courville AB, Guo J, Hall KD. ‘Ad libitum meal energy intake is positively influenced by energy density, eating rate and hyper-palatable food across four dietary patterns’, *Nat Food*, vol. 4, no. 2, pp. 144–147, Jan. 2023, 10.1038/s43016-022-00688-4 10.1038/s43016-022-00688-4 37117850

[82] Lane MM et al. Jun., ‘Ultra-Processed Food Consumption and Mental Health: A Systematic Review and Meta-Analysis of Observational Studies’, *Nutrients*, vol. 14, no. 13, p. 2568, 2022, 10.3390/nu14132568 10.3390/nu14132568 PMC9268228 35807749

[83] Firth J, Gangwisch JE, Borsini A, Wootton RE, Mayer EA. ‘Food and mood: how do diet and nutrition affect mental wellbeing?’, Jun. 2020, 10.1136/bmj.m2382 10.1136/bmj.m2382 PMC7322666 32601102

[84] Morales-Berstein F et al. Mar., ‘Ultra-processed foods, adiposity and risk of head and neck cancer and oesophageal adenocarcinoma in the European Prospective Investigation into Cancer and Nutrition study: a mediation analysis’, *Eur J Nutr*, vol. 63, no. 2, pp. 377–396, 2024, 10.1007/s00394-023-03270-1 10.1007/s00394-023-03270-1 PMC10899298 37989797

[85] Arenas-Jal M, Suñé-Negre JM, Pérez-Lozano P, García-Montoya E. ‘Trends in the food and sports nutrition industry: A review’, *Critical Reviews in Food Science and Nutrition*, vol. 60, no. 14, pp. 2405–2421, Aug. 2020, 10.1080/10408398.2019.1643287 10.1080/10408398.2019.1643287 31352832

[86] Astrup A, Monteiro CA. Does the concept of ultra-processed foods help inform dietary guidelines, beyond conventional classification systems? Debate consensus. Am J Clin Nutr. Oct. 2022;116(6):nqac230. 10.1093/ajcn/nqac230. 10.1093/ajcn/nqac230 36253965

[87] Petrus RR, do Amaral Sobral PJ, Tadini CC, Gonçalves CB. ‘The NOVA classification system: A critical perspective in food science’, *Trends in Food Science & Technology*, vol. 116, pp. 603–608, Oct. 2021, 10.1016/j.tifs.2021.08.010

[88] Sadler CR, Grassby T, Hart K, Raats MM, Sokolović M, Timotijevic L. Even We Are Confused: A Thematic Analysis of Professionals’ Perceptions of Processed Foods and Challenges for Communication. Front Nutr. Feb. 2022;9:826162. 10.3389/fnut.2022.826162. 10.3389/fnut.2022.826162 PMC8904920 35284464

[89] Monteiro CA, Cannon G, Moubarac J-C, Levy RB, Louzada MLC, Jaime PC. Ultra-processing. An odd appraisal. Public Health Nutr. Feb. 2018;21(3):497–501. 10.1017/S1368980017003287. 10.1017/S1368980017003287 PMC10260853 29122052

[90] Food D, Committee O. ‘House of Lords - Recipe for health: a plan to fix our broken food system - Food, Diet and Obesity Committee’. Accessed: Aug. 22, 2025. [Online]. Available: https://publications.parliament.uk/pa/ld5901/ldselect/ldmfdo/19/1902.htm

[91] ‘A UK government food strategy for England’, Accessed GOVUK. Aug. 22, 2025. [Online]. Available: https://www.gov.uk/government/publications/a-uk-government-food-strategy-for-england

[92] Food and Agriculture Organisation of the United Nations. ‘Transforming food and agriculture through a systems approach’. Accessed: Aug. 26, 2025. [Online]. Available: https://openknowledge.fao.org/items/89972635-5a5d-4698-80ec-288be998bc12

[93] ‘A healthy life’, nesta. Accessed: Aug. 22, 2025. [Online]. Available: https://www.nesta.org.uk/healthy-life/

[94] ‘Nourishing Britain. a political manual for improving the nation’s health’, nesta. Accessed: Aug. 22, 2025. [Online]. Available: https://www.nesta.org.uk/report/nourishing-britain/

[95] Hounkpatin H, Goronga T, Chabi SM, Yedenou A. ‘The politics of plenty: why food insecurity persists in a world of abundance’, Aug. 2025, 10.1136/bmj.r1700 10.1136/bmj.r1700 40789605

[96] Adams J, Mytton O, White M, Monsivais P. Why Are Some Population Interventions for Diet and Obesity More Equitable and Effective Than Others? The Role of Individual Agency. PLoS Med. Apr. 2016;13(4):e1001990. 10.1371/journal.pmed.1001990. 10.1371/journal.pmed.1001990 PMC4821622 27046234

[97] Adams J. ‘Food marketing works. What next for the public health community?’, *British Journal of Nutrition*, vol. 133, no. 6, pp. 721–724, Mar. 2025, 10.1017/S0007114524001818 10.1017/S0007114524001818 40518856

[98] Patay D, Reeve E, Thow AM, Baker P, Farrell P. ‘Whole-of-food system governance for transformative change’, *Nat Food*, vol. 6, no. 7, pp. 636–640, Jul. 2025, 10.1038/s43016-025-01196-x 10.1038/s43016-025-01196-x 40646351

[99] Kaur A, Scarborough P, Matthews A, Payne S, Mizdrak A, Rayner M. How many foods in the UK carry health and nutrition claims, and are they healthier than those that do not? Public Health Nutr. Apr. 2016;19(6):988–97. 10.1017/S1368980015002104. 10.1017/S1368980015002104 PMC4825057 26156809

[100] Oostenbach LH, Slits E, Robinson E, Sacks G. ‘Systematic review of the impact of nutrition claims related to fat, sugar and energy content on food choices and energy intake’, *BMC Public Health*, vol. 19, no. 1, p. 1296, Oct. 2019, 10.1186/s12889-019-7622-3 10.1186/s12889-019-7622-3 PMC6794740 31615458

[101] Alsubhi M, Blake M, Nguyen T, Majmudar I, Moodie M, Ananthapavan J. Consumer willingness to pay for healthier food products: A systematic review. Obes Rev. Jan. 2023;24(1):e13525. 10.1111/obr.13525. 10.1111/obr.13525 PMC10909406 36342169

[102] Gressier M et al. Mar., ‘The effectiveness of mandatory v. voluntary food reformulation policies: a rapid review’, *British Journal of Nutrition*, vol. 133, no. 6, pp. 737–750, 2025, 10.1017/S0007114524001326 10.1017/S0007114524001326 PMC12169955 40518854

[103] Ronit K, Jensen JD. ‘Obesity and industry self-regulation of food and beverage marketing: a literature review’, *Eur J Clin Nutr*, vol. 68, no. 7, pp. 753–759, Jul. 2014, 10.1038/ejcn.2014.60 10.1038/ejcn.2014.60 24713622

